# Evaluation of a Respiratory Syncytial Virus Subunit Vaccine Candidate in IgA-Deficient Mice: Insights into the Role of IgA in Vaccine-Induced Immunity and Protection

**DOI:** 10.3390/vaccines14010097

**Published:** 2026-01-20

**Authors:** Liliana Gonzalez Gonzalez, Mina Zhiani, Jourdan Witt, Sylvia van Drunen Littel-van den Hurk

**Affiliations:** 1 Vaccine and Infectious Disease Organization, University of Saskatchewan, Saskatoon, SK S7N 5E3, Canada; 2 School of Public Health, University of Saskatchewan, Saskatoon, SK S7N 2Z4, Canada; 3 Biochemistry, Microbiology and Immunology, University of Saskatchewan, Saskatoon, SK S7N 5E5, Canada

**Keywords:** RSV, intranasal vaccine, tFrsc/TriAdj, IgA^−^/^−^ mice, mucosal immunity, systemic immunity

## Abstract

Background/Objectives: Respiratory Syncytial Virus (RSV) causes severe disease in infants, the elderly, and immunocompromised individuals, with reinfections linked to poor induction of durable mucosal immunoglobulin A (IgA). We investigated the role of IgA in immunity and protection induced by a RSV subunit vaccine candidate, tFrsc/TriAdj, which consists of a truncated RSV fusion protein (tFrsc) with a tri-component adjuvant (TriAdj). Methods: Wild-type (IgA^+^/^+^) and IgA-deficient (IgA^−^/^−^) BALB/c mice were immunized intranasally and subsequently challenged with RSV. Results: Vaccination with tFrsc/TriAdj induced robust systemic and mucosal IgG, and high lung and serum neutralizing antibodies, in both IgA^+^/^+^ and IgA^−^/^−^ mice. As expected, IgA^−^/^−^ mice lacked IgA and exhibited modest reductions in nasal IgG compared to IgA^+/+^ mice following challenge, correlating to failure to clear RSV from the upper respiratory tract. In contrast, viral replication in the lungs was fully suppressed in both genotypes, indicating that IgG alone was sufficient for lower respiratory tract protection. Isotype analysis revealed diminished Th1-associated IgG2a and elevated IgG1 across mucosal and systemic compartments in IgA^−^/^−^ mice, suggesting a Th2 bias. Flow cytometric analysis confirmed reduced recruitment of IFN-γ^+^ CD4^+^ T cells in the lungs of immunized IgA^−^/^−^ mice. Interestingly, IL-17 production and numbers of IL-17^+^ CD4^+^ T cells in the lungs were increased, suggesting an enhanced Th17 response. Furthermore, IgA-deficient mice displayed reduced splenic IgG^+^ B cell populations, which is also a novel observation. Conclusions: Collectively, these findings demonstrate that although tFrsc/TriAdj confers lower airway protection in the absence of IgA, vaccine-induced IgA is critical for upper airway protection, Th1/balanced immune responses, and optimal B cell responses.

## 1. Introduction

Respiratory syncytial virus (RSV) is a leading cause of acute lower respiratory tract (LRT) infections in infants, older adults, and immunocompromised individuals [1]. Infection does not induce durable immunity, allowing for frequent reinfections throughout life. In 2019, RSV was responsible for an estimated 33 million LRT infections and 100,000 deaths globally in children under five, with a significant economic burden in both pediatric and elderly populations [2,3,4]. Recently, three vaccines were approved for use in older adults: Arexvy (GSK), Abrysvo (Pfizer), and mRESVIA (Moderna) [5,6]. These vaccines are administered intramuscularly, a route that primarily induces systemic IgG responses. In the context of mucosal immunity, secretory antibodies play a key role in protecting against viral infection. Particularly, IgA, the predominant antibody isotype at mucosal surfaces, interferes with viral entry in the airways and is secreted at levels exceeding those of IgG [7]. IgA plays a central role in early viral neutralization by blocking attachment and entry without inducing inflammation [7,8].

The protective capacity of IgA has been demonstrated through passive administration of RSV-specific monoclonal IgA to the nasopharyngeal mucosa, which effectively prevented viral infection [9]. In the context of RSV infection, higher nasal IgA more strongly correlates with reduced viral load and infection severity than serum antibody levels, highlighting its importance in early containment [10,11,12]. However, RSV infection is characterized by poor IgA responses, rapid loss of IgA^+^ memory B cells, minimal upregulation of activation-induced cytidine deaminase (AID) and IgA-specific class-switch cytokines, and a rapid decline of antibody-secreting cells (ASCs) in the nasal-associated lymphoid tissue (NALT) [10,13,14,15,16,17]. This contrasts with other respiratory viruses, such as influenza, which induces durable systemic and mucosal responses, including strain-specific nasal IgA and IgA^+^ memory B cells [17,18]. These findings suggest that the high rate of RSV reinfection may stem from its inability to elicit sufficient IgA responses and IgA-secreting memory B cells, thereby limiting the establishment of durable mucosal immunity.

Mucosal vaccination strategies, such as intranasal (IN) immunization, aim to overcome these limitations by targeting the NALT, a key inductive site for upper respiratory immunity and RSV entry [19]. For IN vaccines, the NALT supports both Th1- and Th2-type responses, IgA class switching, affinity maturation, and the generation of memory IgA^+^ B cells [20,21]. Moreover, unlike systemic vaccines, mucosal vaccines have the potential to elicit immune responses that exceed those generated by natural infection and can induce both local and systemic immunity and promote long-term protection [22]. This has been demonstrated by the IN RSV vaccine candidate, tFrsc/TriAdj [23,24,25], which consists of a truncated version of the RSV F protein (tFrsc) formulated with a combination adjuvant (TriAdj) consisting of polyinosinic:polycytidylic acid (polyI:C), a host defense regulatory peptide (IDR1002), and the water-soluble polymer poly[di(sodiumcarboxylatoethylphenoxy) phosphazene] (PCEP). tFrsc/TriAdj has shown strong preclinical efficacy in cotton rats and BALB/c mice, where a single dose induced robust RSV-specific IgA, IgG1, and IgG2a, neutralizing titers, and CD8^+^ effector T cells [16,24,26,27]. Vaccination also enhanced AID expression, IgA class-switching cytokines, and promoted a greater abundance of local IgA-secreting memory B cells [16]. Importantly, a single dose conferred complete protection against RSV challenge, with a Th1-skewed response and durable immunity lasting at least five months [24,26,27]. However, the specific role IgA plays in tFrsc/TriAdj-induced immunity remains undefined.

To address this, we investigated the protective mechanisms of tFrsc/TriAdj in BALB/c mice lacking IgA (IgA^−^/^−^), generated through targeted deletion of the IgA region and part of the constant region of the IgA heavy chain. These mice lack IgA and IgA^+^ B cells but show normal lymphocyte development [28]. IgA^−^/^−^ mice have shown variable protection in other viral models, suggesting a pathogen-dependent role for IgA [29,30,31,32,33]. We hypothesized that the induction of IgA by tFrsc/TriAdj is necessary to establish effective mucosal immunity against RSV and that its absence would lead to reduced protection and altered immune polarization. IgA^−^/^−^ and IgA^+^/^+^ BALB/c mice were INimmunized with tFrsc/TriAdj and then challenged with RSV. While both genotypes developed robust tFsc-specific IgG responses and experienced complete lung protection, IgA^−^/^−^ mice produced lower nasal IgG and experienced impaired clearance of nasal virus. The IgA-deficient mice also developed lower numbers of splenic IgG^+^ B cells and showed altered immune polarization, including lower IgG2a and IFN-γ^+^ T cells, along with a trend towards enhanced Th17 T cells. These results underscore the essential role of vaccine-induced IgA in upper airway protection against RSV and in maintaining balanced immunity.

## 2. Materials and Methods

### 2.1. IgA Knockout Mice

IgA^−^/^−^ BALB/c mice were obtained from Dr. Dennis Metzger (Albany Medical College, Albany, NY, USA). These mice were generated by deleting the IgA switch region and part of the constant region, resulting in the absence of IgA-producing B cells and undetectable levels of IgA, as described previously [30]. Experiments were conducted in both male and female IgA^−^/^−^ mice aged 7 to 10 weeks, and age-matched IgA^+^/^+^ BALB/c mice (Charles River Laboratories, Wilmington, MA, USA). All procedures were approved by the University Animal Care Committee at the University of Saskatchewan in accordance with the standards of the Canadian Council on Animal Care. All animals were cared for under veterinary surveillance.

### 2.2. Virus and Vaccine Formulation

The RSV A2 strain (American Type Culture Collection) was generated in human epithelial type 2 (HEp-2, ATCC, Manassas, VA, USA) cells. The RSV tFrsc protein was prepared as described previously [25]. Briefly, HEK293T cells were transfected with an episomal vector expressing RSV F protein without the cleavage site or transmembrane domain and with HIS tag (tFrsc). The tFrsc protein was purified using TALON Superflow resin (Clontech, Mountain View, CA, USA) according to the manufacturer’s instructions. The tFrsc protein was formulated with LMW Poly(I:C) (InvivoGen, San Diego, CA, USA), IDR-1002 (VQRWLIVWRIRK, Genscript, Piscataway, NJ, USA), and PCEP (Idaho National Laboratory, Idaho Falls, ID, USA) [23]. Poly(I:C), a synthetic double-stranded RNA analog, stimulates type I IFN production and promotes Th1-type responses [34]. IDR-1002 is an innate defense regulator peptide that mediates immune cell recruitment to infection/immunization sites [35]. PCEP is a water-soluble immunostimulatory polymer that elicits Th1/Th2 responses, enhances IgA, and functions as an antigen delivery system through antigen complex formation and presentation [36,37].

### 2.3. Immunization and RSV Challenge

IgA^+^/^+^ and IgA^−^/^−^ BALB/c mice were immunized twice IN under light anesthesia, at a three-week interval, with 10 μL per nostril (total volume 20 μL) of PBS or vaccine containing 1 μg of tFrsc protein, 10 μg Poly(I:C), 20 μg IDR-1002, and 10 μg PCEP. An additional group of mice from each genotype received PBS IN as unchallenged controls in the protection study. Male and female 7- to 10-week-old mice were used, with eight mice per group and equivalent numbers of each sex. Two weeks post-secondary immunization, half of the mice from each genotype, group, and sex were euthanized, and tissues were collected. The remaining half of the mice were IN inoculated with RSV strain A2 (5 × 10^5^ PFU/50 µL) three weeks after secondary immunization. The additional PBS-treated groups were left unchallenged. Four days after the RSV challenge, mice were euthanized, and tissues were collected.

### 2.4. Serum, Nasal Wash, and BAL Collection

Serum samples were collected prior to and after each immunization, as well as after RSV challenge. Nasal wash fluid, bronchoalveolar lavage fluid (BAL), lungs, and spleen were collected on necropsy. Nasal wash fluid was collected by instilling minimum essential medium (MEM, Gibco-Invitrogen, Waltham, MA, USA) supplemented with 10 mM HEPES buffer (Gibco-Invitrogen, Waltham, MA, USA), 2% heat-inactivated fetal bovine serum (FBS, Sigma-Aldrich, Burlington, MA, USA) and 1% antibiotic–antimycotic (100×, Gibco-Invitrogen) into the nasal cavity and stored at −80 °C. Prior to removal, both lungs were washed with ice-cold PBS supplemented with 2% FBS, 1% antibiotic–antimycotic (100×), 3 mM ethylenediaminetetraacetic acid, pH 8.0 (EDTA), and the lavage was collected. BALs were clarified by centrifugation at 800× *g* for 10 min at 4 °C and stored at −20 °C.

### 2.5. Lung Processing and Preparation of Single-Cell Suspensions of Lung and Spleen

Lungs were processed in one of three ways. Lung-homogenate supernatants were prepared as described previously [38]. Briefly, lungs were homogenized using a Mini-Beadbeater (BioSpec Products, Bartlesville, OK, USA) in tubes containing 2.4 mm zirconia microbeads (BioSpec Products) and the same medium used for nasal wash collection. The homogenates were clarified for 1 min at 10,000× *g*, and the supernatants were collected and stored at −80 °C. Lung-fragment cultures (LFCs) were prepared as described previously [38]. Briefly, multi-lobed lungs were cut into small pieces and cultured in RPMI 1640 medium (Gibco-Invitrogen) supplemented with 10% heat-inactivated FBS, 10 mM HEPES buffer, 1% non-essential amino acids (100×, Gibco-Invitrogen), 1 mM sodium pyruvate (Gibco-Invitrogen), 2 mM L-glutamine (Fisher Scientific, Waltham, MA, USA), 1% penicillin–streptomycin (10,000 U/mL, Gibco-Invitrogen), and 50 µg/mL gentamicin (Gibco-Invitrogen) at 37 °C. After a 5-day incubation, supernatants were clarified, and cell-free supernatants were stored at −80 °C.

For single-cell suspensions, lungs were collected in gentleMACS C tubes (Miltenyi Biotec, Bergisch Gladbach, North Rhine-Westphalia, Germany) [16]. Lungs were processed in a gentleMACS dissociator (Miltenyi Biotec) (program m_lung_01), followed by a 30 min incubation with collagenase from Clostridium histolyticum, Type IA to 0.5 mg/mL (Sigma-Aldrich) and deoxyribonuclease I from bovine pancreas, Type IV to 50 μg/mL (DNAse; Sigma-Aldrich) at 37 °C and an additional dissociation step (program m_spleen_01). Red blood cells (RBCs) were lysed in 1x RBC lysis buffer (eBioscience, San Diego, CA, USA). Lung cells were washed in ice-cold PBS supplemented with 0.5% bovine serum albumin (BSA, Sigma Aldrich) and 2 mM EDTA before straining through 70 μm nylon mesh (Corning Incorporated, Corning, NY, USA). Spleens were cut into pieces and collected in gentleMACS C tubes containing RPMI 1640 medium supplemented with 10 mM HEPES, 1% non-essential amino acids (100×), 1 mM sodium pyruvate, 2 mM L-glutamine, 1% antibiotic–antimycotic (100×). Spleens were processed in the gentleMACS dissociator (program m_spleen_01). Supernatants were strained through a 40 μm nylon mesh (Corning Incorporated) before lysing RBCs and resuspending in supplemented RPMI 1640 medium after centrifugation. Cells were passed through a 40 μm nylon mesh to generate single-cell suspensions. Lung and spleen cells were resuspended in 2 mL of RPMI 1640 medium supplemented with 10% heat-inactivated FBS, 10 mM HEPES buffer, 1% non-essential amino acids (100×), 1 mM sodium pyruvate, 2 mM L-glutamine, 1% penicillin–streptomycin (10,000 U/mL) and 0.05 mM 2-mercaeptoethanol (Sigma-Aldrich) and counted with a Particle Count and Size Analyzer (Beckman Coulter Life Sciences, Indianapolis, IN, USA).

### 2.6. Cell Culture, Virus Titration, and Virus-Neutralization Assay

HEp-2 cells were maintained in MEM supplemented with 10% FBS, 10 mM HEPES buffer, 1% penicillin–streptomycin (10,000 U/mL), and 1% antibiotic–antimycotic solution (100×). Virus titration assays were performed as described previously [23]. Briefly, clarified supernatants from homogenized lung, BAL, and nasal wash samples were subjected to ten-fold serial dilutions in supplemented MEM and added to Hep-2 cells. Supernatants were removed after a 2 h incubation at 37 °C and overlaid with a 1:1 mixture of 1.6% low-gelling temperature agarose (Sigma-Aldrich) and 2X MEM (Temin’s modification; Gibco-Invitrogen). The overlay medium was removed after five days at 37 °C, and the cells were fixed with a 4:1 mixture of ethanol and acetic acid and stained with 0.5% Crystal Violet. Viral titers were expressed as plaque-forming units (PFU) per gram of lung tissue or per mL of BAL or nasal wash fluid.

RSV-specific neutralization titers were determined by plaque reduction assays. Sera, LFCs, and nasal washes were serially diluted in 96-well flat-bottom plates (Thermo Scientific) and incubated with 400 PFU/well of RSV strain A2 for 1.25 h at 37 °C. Subsequently, the sample–virus mixtures were transferred to duplicate HEp-2 cell monolayers. After incubation for four days at 37 °C, the cells were fixed and stained as described above. Virus-neutralizing titers were defined as the highest dilution at which ~50% of the cell monolayer remained protected.

### 2.7. Antibody ELISAs

RSV tFrsc-specific IgG, IgG1, IgG2a, and IgA ELISAs were performed as described previously [23]. Briefly, 96-well microtiter plates (Fisher Scientific) were coated with tFrsc protein overnight at 4 °C. Four-fold serially diluted samples were added to the tFrsc-coated plates and incubated overnight at 4 °C. Bound tFrsc-specific antibodies were detected with biotin-conjugated goat anti-mouse IgA (Southern Biotech, Birmingham, AL, USA), biotin-conjugated goat anti-mouse IgG (Invitrogen), biotin-conjugated goat anti-mouse IgG1 (Southern Biotech), or biotin-conjugated goat anti-mouse IgG2a (Southern Biotech), followed by alkaline phosphatase (AP)-conjugated streptavidin (Jackson ImmunoResearch, West Grove, PA, USA) and developed with *p*-nitrophenyl phosphate (pNPP; Sigma-Aldrich) substrate. Absorbance was measured at 405 nm with a 490 nm reference using a BioTek 800 TS Absorbance Microplate Reader (Agilent Products, Santa Clara, CA, USA).

### 2.8. ELISPOTs

T and B cell ELISPOT assays were performed as described previously [16,38]. Briefly, for T cell ELISPOTS, 96-well Multiscreen-HA plates (Millipore Burlington, MA, USA) were coated overnight at 4 °C with murine IFN-γ- (BD Pharmigen, San Jose, CA, USA), IL-5- (BD Pharmigen), or IL-17A- (BioLegend, San Diego, CA, USA) specific monoclonal antibodies. Splenocytes (10^6^ cells/well) were stimulated with tFrsc protein, Concanavalin A (Sigma-Aldrich), or culture medium, and added in triplicate to the coated plates. After overnight incubation at 37 °C, the plates were washed and incubated with biotinylated rat anti-mouse IFN-γ- (BD Pharmigen), IL-5- (BD Pharmigen), or IL-17A (Biolegend)-specific polyclonal antibody. For B cell ELISPOTs, plates were coated overnight with tFrsc protein or PBS. Splenocytes or lung cells were added in triplicate, and the plates were incubated overnight at 37 °C, followed by washing and blocking with 1% BSA. Subsequently, plates were incubated with biotinylated goat anti-mouse IgA (Southern Biotech) or anti-mouse IgG (Invitrogen) polyclonal antibody. Finally, all plates were incubated with AP-conjugated streptavidin, and spots representing IFN-γ-, IL-5-, IL17-, IgA-, and IgG-secreting cells were visualized with BCIP/NBT (Sigma-Aldrich) substrate. Results are expressed as the difference between the number of cytokine- or antibody-secreting cells per 10^6^ cells in tFrsc protein-stimulated wells and the number of cytokine- or antibody-secreting cells per 10^6^ cells in medium-treated wells.

### 2.9. Cytokine ELISAs

Splenocytes and lung cells were restimulated in vitro with the tFrsc protein in 96-well round bottom tissue culture plates (Fisher Scientific) in RPMI 1640 medium supplemented with 10% heat-inactivated FBS, 10 mM HEPES buffer, 1% non-essential amino acids (100×), 1 mM sodium pyruvate, 1 mM L-glutamine, 1% penicillin–streptomycin (10,000 U/mL), and 0.05 mM 2-mercaeptoethanol (Sigma-Aldrich). Cells (10^6^ cells/well) were incubated with culture medium containing tFrsc protein and NA/LE hamster anti-mouse CD28 (BD Pharmigen, San Jose, CA, USA) and/or NA/LE rat anti-mouse CD49d (BD Pharmingen), or with Con A. After 72 h at 37 °C, plates were centrifuged at 1583× *g* for 10 min, and the cell-free supernatants were collected and stored at −80 °C.

Nunc-Immuno MaxiSorp plates (Thermo Scientific) were incubated overnight at 4 °C with rat anti-mouse IFN-γ (BD Pharmigen), IL-17A (BioLegend), or IL-5 (BD Pharmingen). After overnight incubation, plates were blocked with 1% BSA and incubated with tFrsc protein-, medium- or ConA-treated cell supernatants. Subsequently, plates were incubated with biotinylated rat anti-mouse IFN-γ (BD Pharmigen), rat anti-mouse IL-5 (BD Pharmingen), or rat anti-mouse IL-17A (BioLegend)-specific antibodies, followed by AP-conjugated streptavidin, and developed with pNPP substrate. Absorbance was measured at 405 nm with a 490 nm reference by using a BioTek 800 TS Absorbance Microplate Reader. Results are presented as the difference in optical density (OD) between tFrsc protein-stimulated wells and medium control wells for each individual mouse. Cytokine concentrations were quantified by extrapolation from standard curves generated using recombinant murine IFN-γ, IL-5, or IL-17A, based on the net OD values (OD_tFrsc_–OD_medium_), and expressed as pg/mL cytokine.

### 2.10. Analysis of tFrsc Protein-Induced CD4^+^ T Cells by Flow Cytometry

Lung and splenocyte single-cell suspensions were treated with RPMI 1640 medium supplemented with 10% heat-inactivated FBS, 10 mM HEPES buffer, 1% non-essential amino acids (100×), 100 mM sodium pyruvate, 2 mM L-glutamine, 1% penicillin–streptomycin (10,000 U/mL), and 0.05 mM 2-mercaptoethanol containing tFrsc protein, hamster anti-mouse CD28, and rat anti-mouse CD49d. After 18 h incubation at 37 °C, cells were incubated with BD GolgiPlug Protein Transport Inhibitor (Brefeldin A; BD Biosciences, Franklin Lakes, NJ, USA) for 6 h. Cells were treated with LIVE/DEAD Fixable Aqua Dead Cell Stain (Gibco-Invitrogen) and Fc receptor blocking TruStain FcX anti-mouse CD16/32 antibody solution (BioLegend), followed by cell surface staining using PE anti-mouse CD45 (BioLegend), FITC anti-mouse CD4 (BioLegend), and PE/Cyanine7 anti-mouse/human CD44 (BioLegend) monoclonal antibodies. Subsequently, cells were fixed and permeabilized using a Cytofix/Cytoperm solution (BD Biosciences). Intracellular cytokine staining was performed using Alexa Fluor 647 Mouse anti-Human IL-17A (IL-17A-AF647; BioLegend) and Brilliant Violet 421 anti-mouse IFN-γ (IFN-g-BV421, Biolegend) fluorochrome-conjugated monoclonal antibodies in Cytofix/Cytoperm solution. Cells were acquired by flow cytometry using a CytoFLEX (Beckman-Coulter Life Sciences), and data were analyzed using FlowJo Software Version 10.10 (BD Biosciences). Cells were gated for lymphocytes, singlets, and live cells, and then analyzed for the indicated markers. Results are expressed as the difference between the percentage of IL-17A^+^CD4^+^CD44^+^ or IFN-γ^+^CD4^+^CD44^+^ T cells in RSV tFrsc protein-stimulated wells and the percentage of IL-17A^+^CD4^+^CD44^+^ or IFN-γ^+^CD4^+^CD44^+^ T cells in medium-stimulated wells.

### 2.11. Statistical Analysis

All data were analyzed using GraphPad PRISM version 9 for Windows (GraphPad Software, Boston, MA, USA). Normal distribution of samples among all groups was examined using the Shapiro–Wilk normality test. If the distribution between the pairs of groups to be compared was found to be normal (alpha = 0.05), then means between said pairs of groups were compared using an unpaired *t*-test to determine the *p*-value. If the distribution of any group to be compared did not pass the normality test, then median ranks between pairs of groups were compared using an unpaired nonparametric the Mann–Whitney U test to determine the *p*-value. Differences were considered significant if *p* < 0.05.

## 3. Results

### 3.1. Humoral Immune Responses and Protection from RSV Infection Following IN tFrsc/TriAdj Immunization in Wild-Type and IgA-Deficient Mice

Vaccination and challenge trials were initially conducted separately in wild-type and IgA^−^/^−^ mice to determine whether the two genetic backgrounds elicited similar or divergent antibody responses. All experimental assays and data analyses were carried out concurrently. The results are representative of multiple animal trials, comprising two to three independent trials per genetic background and immunization condition (vaccination alone or vaccination followed by challenge).

#### 3.1.1. Systemic and Mucosal Humoral Immune Responses Induced by tFrsc/TriAdj

**Figure 1:**
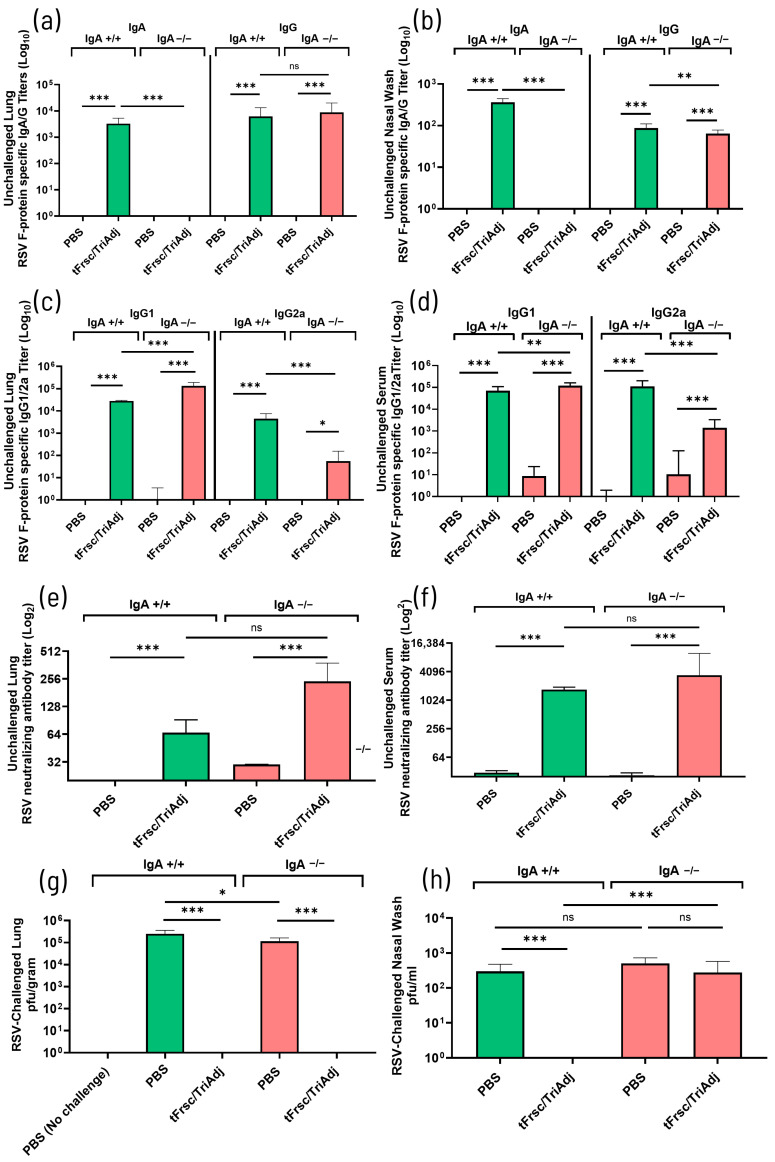
Immune responses to RSV tFrsc protein and virus replication in IgA^+^/^+^ and IgA^−^/^−^ BALB/c mice after two IN immunizations. Mice were immunized twice, three weeks apart, with PBS or tFrsc/TriAdj. To analyze immune responses, mice were euthanized two weeks post-secondary vaccination (**a**–**f**). To analyze protection, mice were IN challenged with 5 × 10^5^ PFU of the RSV A2 strain three weeks post-secondary immunization and euthanized four days post-challenge (**g**,**h**). (**a**) LFC IgA and IgG titers specific for RSV tFrsc protein, (**b**) nasal wash IgA and IgG titers specific for RSV tFrsc protein, (**c**) LFC IgG1 and IgG2a titers specific for RSV tFrsc protein, (**d**) serum IgG1 and IgG2a titers specific for RSV tFrsc protein, (**e**) LFC RSV-neutralizing antibody titers, (**f**) serum RSV-neutralizing antibody titers. Antibody titers were analyzed by ELISA and are expressed as the reciprocal of the highest dilution resulting in a value of two standard deviations above the negative control samples. Viral titers were determined in lung homogenates (**g**) and nasal washes (**h**). Each bar represents the mean RSV titer per gram of lung or milliliter of nasal wash for eight mice per group. Data are presented as median with interquartile range for eight mice per group. Statistical differences between IgA^+^/^+^ and IgA^−^/^−^ groups were assessed following normality testing (Shapiro–Wilk). Data were analyzed using an unpaired nonparametric Mann–Whitney U-test. Differences were considered significant if *p* < 0.05. * *p* < 0.05; ** *p* < 0.01; *** *p* < 0.001; ns = non-significant.

To evaluate the mucosal humoral immune responses induced by tFrsc/TriAdj in IgA-deficient mice, we determined the level of IgA and IgG specific for the tFrsc protein in LFC and nasal wash from vaccinated IgA^+^/^+^ and IgA^−^/^−^ mice obtained two weeks after secondary immunization. Both mouse genotypes developed robust IgG titers after vaccination when compared to mice that received PBS (*p* < 0.001) (Figure 1a,b). There was no significant difference in lung IgG between tFrsc/TriAdj-vaccinated mouse genotypes (Figure 1a). tFrsc/TriAdj induced a lower nasal anti-tFrsc IgG response in IgA^−^/^−^ mice (*p* < 0.01) compared to IgA^+^/^+^ mice (Figure 1b). As expected, high IgA titers were induced in IgA^+^/^+^ mice, while IgA was absent in IgA^−^/^−^ mice.

To define the type of immune response induced in IgA^−^/^−^ mice, we measured local and systemic IgG1 and IgG2a subclass levels specific for the tFrsc protein in the lungs and sera. Lungs of both genotypes immunized with tFrsc/TriAdj had significantly higher IgG1 and IgG2a levels than those of the PBS groups (*p* < 0.001 or *p* < 0.05) (Figure 1c). In the IgA^+/+^ mice, the relative levels of IgG1 and IgG2a, present at a ratio of ≈ 1:1, suggest a balanced immune response. However, IgA^−^/^−^ mice developed a significantly higher IgG1 (*p* < 0.001) and lower IgG2a (*p* < 0.001) response when compared to IgA^+^/^+^ animals. In contrast to IgA^+^/^+^ mice, the IgG1 level was higher than the IgG2a response. Intranasal tFrsc/TriAdj immunization also induced significant levels of tFrsc-specific IgG1 and IgG2a in serum of both IgA^+^/^+^ and IgA^−^/^−^ mice compared to PBS controls (*p* < 0.001). However, consistent with local responses observed in lung tissue, IgA^−^/^−^ mice exhibited elevated IgG1 (*p* < 0.01) and reduced IgG2a (*p* < 0.001) levels, indicating a shift towards a Th2-type response, whereas IgA^+^/^+^ mice maintained a more balanced isotype distribution (Figure 1d).

To assess the functional activity of antibodies generated in IgA^−^/^−^ mice following IN immunization, RSV-neutralizing titers were measured. The lungs of tFrsc/TriAdj-immunized mice from both genotypes exhibited significantly higher neutralizing antibody titers compared to PBS-treated animals (*p* < 0.001), with no differences observed between IgA^+^/^+^ and IgA^−^/^−^ mice (Figure 1e). In serum, both IgA^+^/^+^ and IgA^−^/^−^ mice developed robust neutralizing antibody responses (*p* < 0.001 compared to PBS groups), with comparable antibody levels between mouse genotypes, indicating that systemic immunity was not impaired by the absence of IgA (Figure 1f).

#### 3.1.2. Protection from RSV Challenge

To assess whether the absence of IgA affected protection against RSV, IgA^+^/^+^ and IgA^−^/^−^ mice were IN challenged with RSV three weeks after secondary immunization. RSV was recovered from the lungs of both IgA^+^/^+^ and IgA^−^/^−^ mice in the PBS groups, whereas no virus was detected in the lungs of tFrsc/TriAdj-immunized mice (*p* < 0.001) (Figure 1g). While the difference in RSV titers between PBS-treated IgA^+^/^+^ and IgA^−^/^−^ mice reached statistical significance (*p* < 0.05), the levels were broadly similar, indicating minimal biological relevance. In contrast, viral replication was present in the nasal passages of both PBS- and tFrsc/TriAdj-immunized IgA^−^/^−^ mice, while no virus was detected in tFrsc/TriAdj-immunized IgA^+^/^+^ mice (*p* < 0.001 compared to IgA^+^/^+^ PBS group) (Figure 1h). This indicates incomplete viral clearance in the nasal passages of tFrsc/TriAdj-immunized IgA^−^/^−^ mice, suggesting that mucosal tFrsc-specific IgA may be essential for effective protection in the URT but not the LRT.

### 3.2. Direct Comparison of Immune Responses and Protection from RSV Induced by tFrsc/TriAdj Immunization in Wild-Type and IgA-Deficient Mice

After characterizing humoral mucosal and systemic antibody responses and identifying potential alterations in IgA-deficient mice, we designed a study to also enable analysis and comparison of T and B cell responses. This simultaneous vaccination and challenge study in wild-type and IgA^−^/^−^ mice was performed to allow direct comparison between genotypes and served to validate the findings described above.

#### 3.2.1. Divergent IgG Subclass and Mucosal Antibody Responses in IgA-Deficient Mice Following IN Immunization

**Figure 2:**
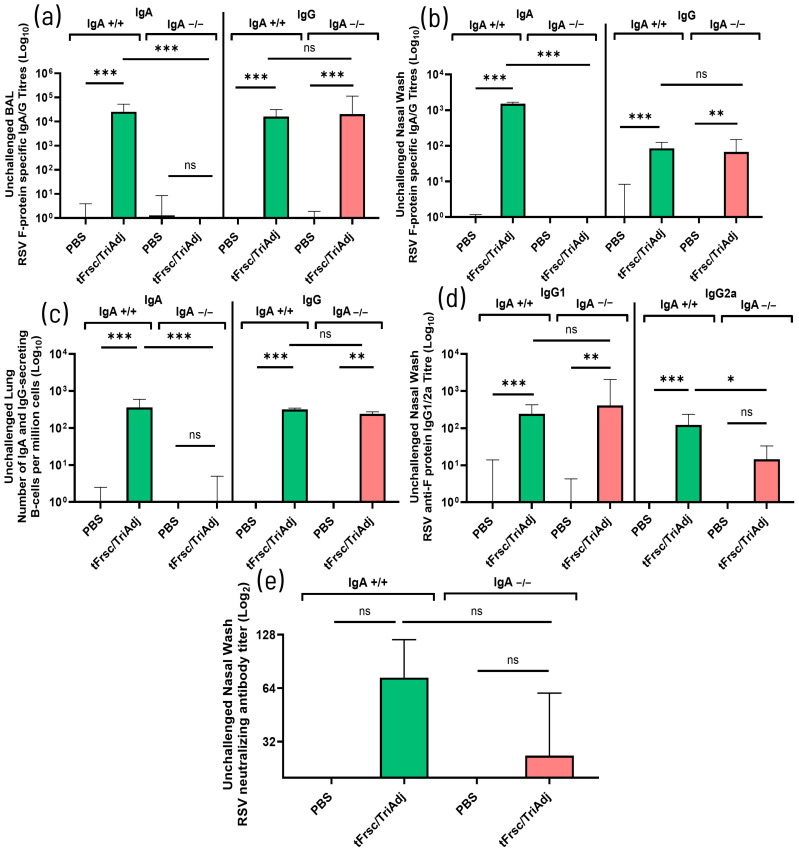
Mucosal humoral immune responses to RSV tFrsc protein in IgA^+^/^+^ and IgA^−^/^−^ BALB/c mice after two IN immunizations. Mice were immunized twice, three weeks apart, with PBS or tFrsc/TriAdj, and were euthanized two weeks post-secondary vaccination. (**a**) BAL IgA and IgG titers specific for RSV tFrsc protein. (**b**) Nasal wash IgA and IgG titers specific for RSV tFrsc protein. (**c**) Lung RSV tFrsc-specific IgA and IgG-secreting B cells. (**d**) Nasal wash IgG1 and IgG2a titers specific for RSV tFrsc protein. (**e**) Nasal wash RSV-neutralizing antibody titers. Antibody titers were analyzed by ELISA and are expressed as the reciprocal of the highest dilution resulting in a value of two standard deviations above the negative control samples. Numbers of tFrsc-specific antibody-secreting B cells were measured by ELISpot. Data are expressed as the difference between the number of B cells in tFrsc-protein-stimulated wells and medium wells. Neutralization titers were defined as the first concentration of antibody required for a 50% reduction in the RSV infectivity in HEp-2 cell cultures. Data are presented as median with interquartile range for eight mice per group. Statistical differences between IgA^+^/^+^ and IgA^−^/^−^ groups were assessed following normality testing (Shapiro–Wilk). Data were analyzed using an unpaired nonparametric Mann–Whitney U-test. Differences were considered significant if *p* < 0.05. * *p* < 0.05; ** *p* < 0.01; *** *p* < 0.001; ns = non-significant.

To confirm that tFrsc/TriAdj induces distinct mucosal humoral responses in wild-type and IgA-deficient mice, we measured tFrsc-specific IgA and IgG titers in BAL and nasal wash samples. As expected, vaccination induced robust tFrsc-specific IgA responses in IgA^+^/^+^ mice across both sample types, while no IgA was detected in IgA^−^/^−^ mice. Prior to challenge, both genotypes showed significantly elevated tFrsc-specific IgG levels following tFrsc/TriAdj vaccination compared to PBS treatment (*p* < 0.01 or *p* < 0.001), with no differences between vaccinated IgA^+^/^+^ and IgA^−^/^−^ mice in either sample type (Figure 2a,b). As expected, antigen-specific IgA-secreting B cells were absent in the lungs of IgA^−^/^−^ mice, while they were induced in tFrsc/TriAdj-immunized IgA^+^/^+^ mice when compared to PBS treatment (*p* < 0.001) (Figure 2c). However, immunization significantly increased tFrsc-specific IgG-secreting B cell responses in both IgA^+^/^+^ and IgA^−^/^−^ mice compared to PBS-treated animals (*p* < 0.01 or *p* < 0.001). No differences in the number of IgG-secreting cells were observed between the two genotypes following immunization, indicating that IgA^−^/^−^ mice did not exhibit a local compensatory antigen-specific B-cell response.

To assess the type of immune response induced locally by tFrsc/TriAdj, we measured tFrsc-specific IgG1 and IgG2a levels in nasal washes. In IgA^+^/^+^ mice, vaccination induced high levels of both IgG1 and IgG2a (*p* < 0.001), whereas IgA^−^/^−^ mice showed a significant increase in IgG1 only (*p* < 0.01) when compared to PBS (Figure 2d). Notably, IgG2a levels were not different from those in the PBS group and were significantly lower in IgA^−^/^−^ mice compared to IgA^+^/^+^ mice (*p* < 0.05), suggesting a shift towards a Th2-type response. These findings are consistent with the IgG subclass profile observed in the lung analysis described above.

To further assess local responses to RSV, we measured tFrsc-specific RSV-neutralizing antibody levels in nasal wash samples. Significant nasal virus-neutralizing antibodies were induced in the IgA^+^/^+^ mice, but not in the IgA^−^/^−^ mice, in comparison to those in PBS-treated mice. Although no significant differences were observed between IgA^+^/^+^ and IgA^−^/^−^ mice, the neutralizing antibody titers tended to be lower in the IgA^−^/^−^ mice (Figure 2e), further suggesting that the neutralizing capacity in the nasal compartment might be reduced in the IgA^−^/^−^ mice. This contrasts with the robust neutralizing response observed in the lungs following immunization, indicating that tFrsc/TriAdj-induced protection is more effective in the LRT than in the upper airway of IgA^−^/^−^ mice.

**Figure 3:**
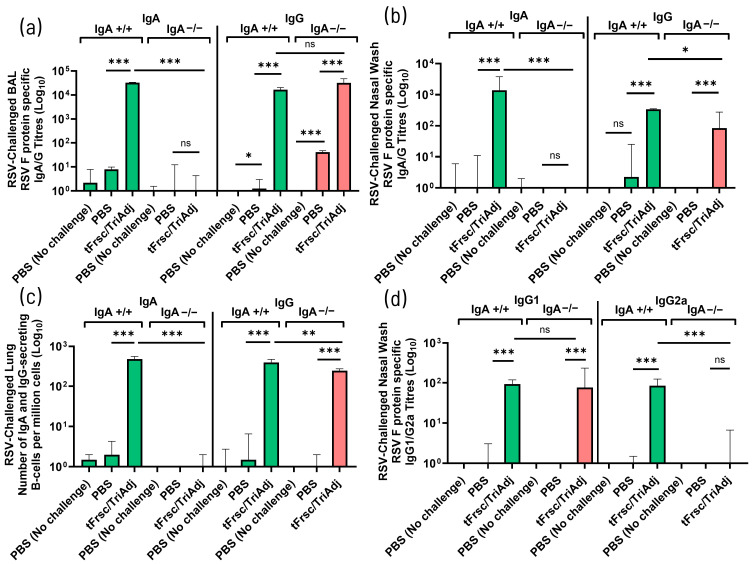
Mucosal humoral immune responses to RSV tFrsc protein in IgA^+^/^+^ and IgA^−^/^−^ BALB/c mice after two IN immunizations followed by RSV challenge. Mice were immunized twice, three weeks apart, with PBS or tFrsc/TriAdj, followed by IN challenge with 5 × 10^5^ PFU of RSV A2 strain three weeks post-secondary immunization and euthanization four days post-challenge. (**a**) BAL IgA and IgG titers specific for RSV tFrsc protein. (**b**) Nasal wash IgA and IgG titers specific for RSV tFrsc protein. (**c**) Lung tFrsc-specific IgA and IgG-secreting B cells. (**d**) Nasal wash IgG1 and IgG2a titers specific for RSV tFrsc protein. Antibody titers were analyzed by ELISA and are expressed as the reciprocal of the highest dilution resulting in a value of two standard deviations above the negative control samples. Numbers of tFrsc-specific antibody-secreting B cells were measured by ELISpot. Data are expressed as the difference between the number of B cells in tFrsc-protein-stimulated wells and medium wells. Values of zero were graphed as one to display all the mice. Data are presented as median with interquartile range for eight mice per group. Statistical differences between IgA^+^/^+^ and IgA^−^/^−^ groups were assessed following normality testing (Shapiro–Wilk). Normally distributed data were analyzed using an unpaired *t*-test, and non-normal data using a Mann–Whitney U-test. Differences were considered significant if *p* < 0.05. * *p* < 0.05; ** *p* < 0.01; *** *p* < 0.001; ns = non-significant.

Post-challenge results largely mirrored those observed prior to RSV exposure (Figure 3a–d). However, notable differences were observed in the URT and LRT. Specifically, IgG levels in the nasal washes of tFrsc/TriAdj-immunized IgA^−^/^−^ mice were significantly lower than those in IgA^+^/^+^ mice (*p* < 0.05) (Figure 3b). Although IgG-secreting B cell responses in the lungs were reduced in IgA^−^/^−^ mice (*p* < 0.01), the reduction appeared to be modest (Figure 3c). Importantly, IgG2a was completely absent in tFrsc/TriAdj-immunized IgA^−^/^−^ mice (*p* < 0.001 compared to IgA^+^/^+^ mice), while IgG1 responses were preserved in both genotypes (Figure 3d). These findings confirm results described in the previous section, showing that tFrsc/TriAdj induces broad mucosal antibody responses in IgA^+^/^+^ mice, while IgA^−^/^−^ mice exhibit a significantly reduced nasal IgG2a response post-challenge consistently across two independent trials.

#### 3.2.2. Altered Systemic IgG2a and B-Cell Responses in IgA-Deficient Mice

**Figure 4:**
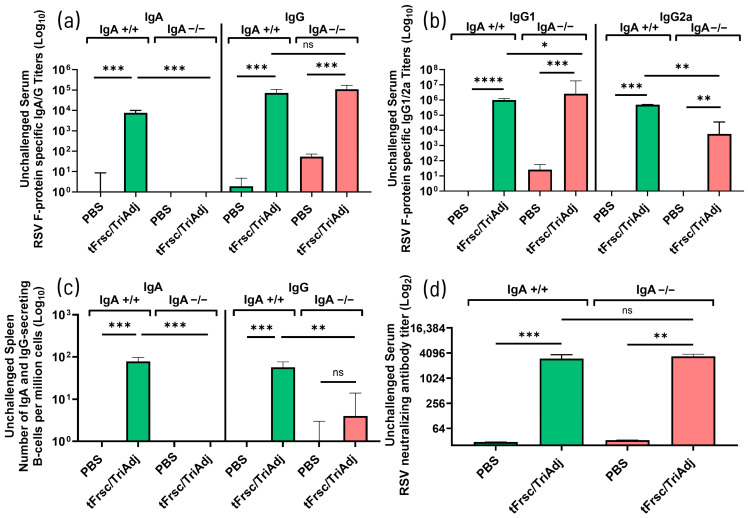
Systemic humoral immune responses to RSV tFrsc protein in IgA^+^/^+^ and IgA^−^/^−^ BALB/c mice after two IN immunizations. Mice were immunized twice, three weeks apart, with PBS or tFrsc/TriAdj and were euthanized two weeks post-secondary vaccination. (**a**) Serum RSV tFrsc-specific IgA and IgG titers. (**b**) Serum RSV tFrsc-specific IgG1 and IgG2a titers. (**c**) Spleen RSV tFrsc-specific IgA and IgG-secreting B cell responses. (**d**) Serum RSV-neutralizing antibody titers. Antibody titers were measured from final serum samples collected at the time of euthanasia. ELISA titers are expressed as the reciprocal of the highest dilution resulting in a value of two standard deviations above the negative control samples. Spleen tissues were used to isolate splenocytes. Numbers of tFrsc-specific antibody-secreting B cells were measured by ELISpot. Data are expressed as the difference between the number of B cells in tFrsc-protein-stimulated wells and medium wells. Neutralization titers were defined as the first concentration of antibody required for a 50% reduction in the RSV infectivity. Data are presented as median with interquartile range for eight mice per group. Statistical differences between IgA^+^/^+^ and IgA^−^/^−^ groups were assessed following normality testing (Shapiro–Wilk). Normally distributed data were analyzed using an unpaired *t*-test, and non-normal data using a Mann–Whitney U-test. Differences were considered significant if *p* < 0.05. * *p* < 0.05; ** *p* < 0.01; *** *p* < 0.001; **** *p* < 0.0001; ns = non-significant.

To further characterize the systemic humoral immune responses induced by IN vaccination with tFrsc/TriAdj, serum antibody levels and splenic antibody-secreting B cell responses were compared between IgA^+^/^+^ and IgA^−^/^−^ mice. Vaccination significantly increased tFsc-specific serum IgG levels in both genotypes (*p* < 0.001), with no significant difference in IgG titers between IgA^+^/^+^ and IgA^−^/^−^ mice (Figure 4a and Supplementary Materials Figure S1a,b). As expected, serum IgA was undetectable in IgA^−^/^−^ mice, whereas tFrsc/TriAdj-immunized IgA^+^/^+^ mice developed strong tFrsc-specific IgA responses (*p* < 0.001). Furthermore, both strains mounted serum IgG1 and IgG2a responses following immunization (*p* < 0.01, *p* < 0.001, or *p* < 0.0001 compared to PBS). Consistent with previous observations in unchallenged mice, both systemically and in the lungs, IgA^−^/^−^ mice exhibited elevated serum IgG1 (*p* < 0.05) and lower IgG2a levels (*p* < 0.01) compared to IgA^+^/^+^ mice, further indicating a shift toward a Th2-skewed response in the absence of IgA (Figure 4b and Supplementary Materials, Figure S1c,d).

As expected, IgA-secreting B cells were absent in IgA^−^/^−^ mice. In contrast, IgA^+^/^+^ mice exhibited a significant increase in both IgA^+^ and IgG^+^ B cell numbers following tFrsc/TriAdj vaccination (*p* < 0.001 compared to PBS). However, tFrsc/TriAdj-immunized IgA^−^/^−^ mice showed a significantly reduced IgG^+^ B cell response compared to IgA^+^/^+^ mice (*p* < 0.01), with no difference between vaccinated and PBS-treated groups, suggesting impaired B cell activation or survival in the systemic compartment (Figure 4c). Both IgA^+^/^+^ and IgA^−^/^−^ mice immunized with tFrsc/TriAdj exhibited significantly higher serum neutralizing activity compared to their respective PBS controls (*p* < 0.001 and *p* < 0.01, respectively), but no differences were observed between the vaccinated IgA^+^/^+^ and IgA^−^/^−^ mice (Figure 4d).

**Figure 5:**
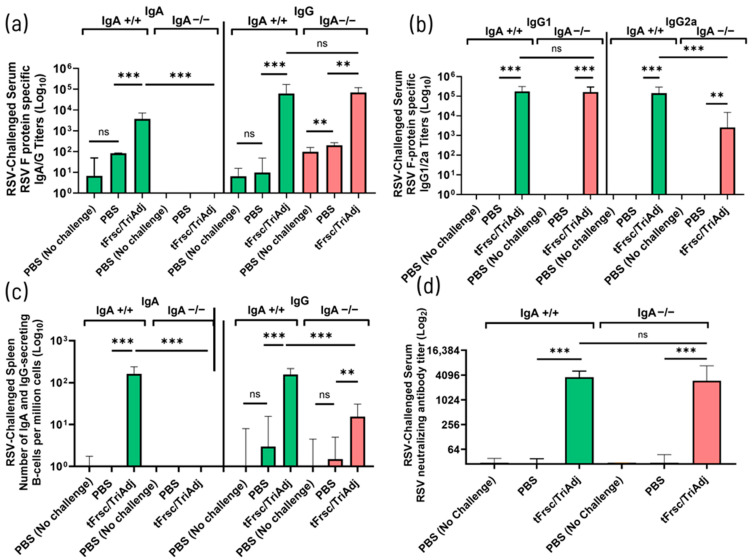
Systemic humoral immune responses to RSV tFrsc protein in IgA^+^/^+^ and IgA^−^/^−^ BALB/c mice after two IN immunizations followed by RSV challenge. Mice were immunized twice, three weeks apart, with PBS or tFrsc/TriAdj, followed by IN challenge with 5 × 10^5^ PFU of RSV A2 strain three weeks post-secondary immunization and euthanization four days post-challenge. (**a**) Serum RSV tFrsc-specific IgA and IgG titers. (**b**) Serum RSV tFrsc-specific IgG1 and IgG2a titers. (**c**) Spleen RSV tFrsc-specific IgA and IgG-secreting B cells. (**d**) Serum RSV-neutralizing antibody titers. Antibody titers were measured from final serum samples collected at the time of euthanasia and were analyzed by subclass-specific ELISA. ELISA titers are expressed as the reciprocal of the highest dilution resulting in a value of two standard deviations above the negative control samples. Spleen tissues were used to isolate splenocytes. Numbers of tFrsc-specific antibody-secreting B cells were measured by ELISpot. Data are expressed as the difference between the number of B cells in tFrsc-protein-stimulated wells and medium wells. Neutralization titers were defined as the first concentration of antibody required for a 50% reduction in the RSV infectivity. Data are presented as median with interquartile range for eight mice per group. Statistical differences between IgA^+^/^+^ and IgA^−^/^−^ groups were assessed following normality testing (Shapiro–Wilk). Normally distributed data were analyzed using an unpaired *t*-test, and non-normal data using a Mann–Whitney U-test. Differences were considered significant if *p* < 0.05. ** *p* < 0.01; *** *p* < 0.001; ns = non-significant.

Systemic antibody responses following RSV challenge were consistent with those observed prior to challenge, confirming that IN tFrsc/TriAdj vaccination induces strong humoral immunity compared to treatment with PBS. tFrsc-specific IgG levels remained significantly elevated in vaccinated IgA^+^/^+^ and IgA^−^/^−^ mice compared to PBS-treated mice (*p* < 0.01 or *p* < 0.001), with no difference between genotypes (Figure 5a and Supplementary Materials, Figure S2a,b). 

Similarly, both genotypes maintained robust tFrsc-specific IgG1 and IgG2a subclass responses, with tFrsc/TriAdj-immunized IgA^−^/^−^ mice exhibiting significantly reduced IgG2a levels (*p* < 0.001) while maintaining IgG1 levels comparable to IgA^+^/^+^ mice (Figure 5b and Supplementary Materials Figure S2c,d). Splenic tFrsc-specific IgG-secreting B cells were significantly elevated in tFrsc/TriAdj-vaccinated mice compared to PBS-treated animals, although responses remained lower in IgA^−^/^−^ mice than in IgA^+^/^+^ mice (*p* < 0.001) (Figure 5c). Robust RSV-neutralizing antibody titers were maintained following challenge and remained comparable between tFrsc/TriAdj-immunized IgA^+^/^+^ and IgA^−^/^−^ mice, while being significantly higher than those in PBS-treated animals (*p* < 0.001) (Figure 5d).

#### 3.2.3. tFrsc/TriAdj-Induced Cytokine Responses and T Cell Recruitment

**Figure 6:**
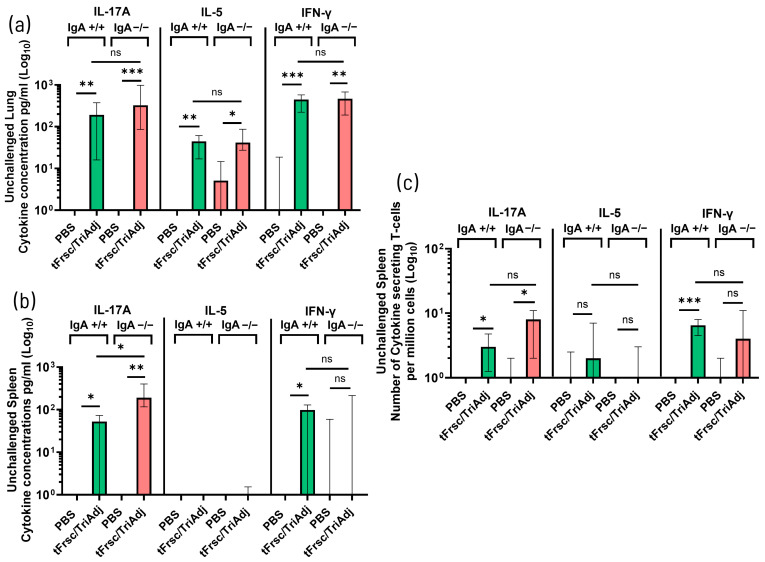
T cell responses to RSV tFrsc protein in IgA^+^/^+^ and IgA^−^/^−^ BALB/c after two IN immunizations. Mice were immunized twice, three weeks apart, with PBS or tFrsc/TriAdj and were euthanized two weeks post-secondary vaccination. Lungs and spleens were used to isolate single lung cells and splenocytes, respectively. For assessment of cytokine concentrations, single-cell isolates were incubated ex vivo with tFrsc protein or medium, anti-CD28, and anti-CD49d antibodies for 72 h. Supernatants were used to determine cytokine concentrations by ELISA. tFrsc-specific Th1-type (IFN-γ), Th2-type (IL-5), and Th17-type (IL-17) cytokine concentrations (pg/mL) for lungs (**a**) and spleens (**b**), and spleen cytokine-secreting T cell responses (**c**). Data are expressed as the difference between the cytokine concentrations in tFrsc-stimulated wells and medium wells for 8 mice per group. Numbers of spleen tFrsc-specific cytokine-secreting T cells were measured by ELISpot. Data are expressed as the difference between the number of T cells in tFrsc-stimulated wells and medium wells. Data are shown as the average of three replicates for 8 mice per group. Data are presented as median with interquartile range. Statistical differences between IgA^+^/^+^ and IgA^−^/^−^ groups were assessed following normality testing (Shapiro–Wilk). Normally distributed data were analyzed using an unpaired *t*-test, and non-normal data using a Mann–Whitney U-test. Differences were considered significant if *p* < 0.05. * *p* < 0.05; ** *p* < 0.01; *** *p* < 0.001; ns = non-significant.

To assess vaccine-induced T cell responses, cytokine production in the lungs and spleen was evaluated following in vitro restimulation with tFrsc protein, along with the frequency of cytokine-secreting T cells in the spleen. Restimulated lung cells from both tFrsc/TriAdj-vaccinated IgA^+^/^+^ and IgA^−^/^−^ mice produced significantly more IL-17, IL-5, and IFN-γ compared to those from PBS-immunized animals (*p* < 0.05, *p* < 0.01, or *p* < 0.001), with no notable genotype differences (Figure 6a). Vaccination with tFrsc/TriAdj induced significantly elevated IL-17A production by splenocytes in both IgA^+^/^+^ and IgA^−^/^−^ mice compared to PBS-treated animals (*p* < 0.05 and *p* < 0.01, respectively), with IgA^−^/^−^ mice producing higher IL-17A levels than IgA^+^/^+^ counterparts (*p* < 0.05) (Figure 6b). IFN-γ was significantly increased only in vaccinated IgA^+^/^+^ mice (*p* < 0.05), with a trend toward reduced production in IgA^−^/^−^ mice, while IL-5 was undetectable across all groups. 

Analysis of splenic cytokine-secreting T cells revealed a significant increase in IL-17-secreting T cells in both tFrsc/TriAdj-vaccinated IgA^+^/^+^ and IgA^−^/^−^ mice compared to PBS-treated animals (*p* < 0.05), while IFN-γ-secreting T cells were significantly elevated only in IgA^+^/^+^ mice (*p* < 0.001) (Figure 6c). Together, splenic cytokine secretion and T cell analyses suggest a trend toward increased IL-17 and IL-17^+^ T cells, alongside reduced IFN-γ and IFN-γ^+^ T cells in IgA^−^/^−^ mice.

**Figure 7:**
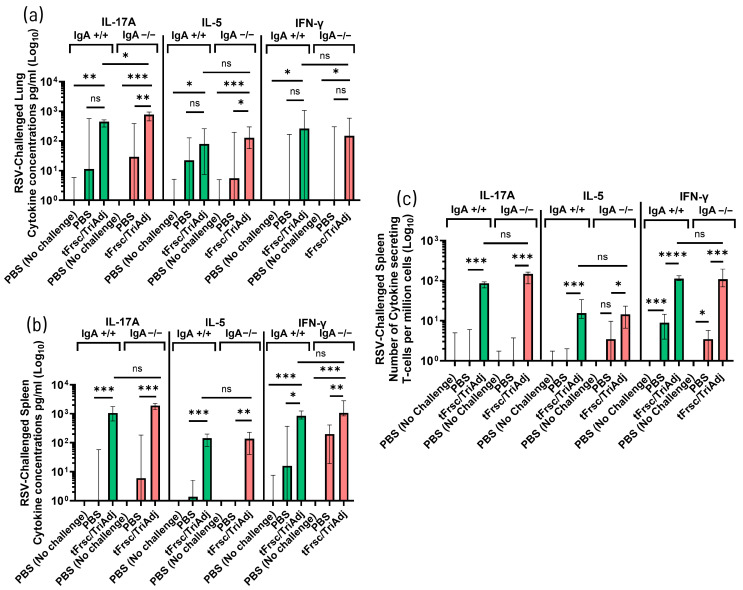
T cell responses to RSV tFrsc protein in IgA^+^/^+^ and IgA^−^/^−^ BALB/c mice after two IN immunizations followed by RSV challenge. Mice were immunized twice, three weeks apart, with PBS or tFrsc/TriAdj, followed by IN challenge with 5 × 10^5^ PFU of RSV A2 strain three weeks post-secondary immunization and euthanization four days post challenge. Lungs and spleens were used to isolate single lung cells and splenocytes, respectively. RSV tFrsc-specific Th1-type (IFN-γ), Th2-type (IL-5), and Th17-type (IL-17) cytokine concentrations (pg/mL) for lungs (**a**) and spleens (**b**) and cytokine-secreting splenic T cells (**c**). For assessment of cytokine concentrations, single-cell isolates were incubated ex vivo with tFrsc protein or medium, anti-CD28, and anti-CD49d antibodies for 72 h. Supernatants were used to determine cytokine concentrations by ELISA. Data are expressed as the difference between the cytokine concentrations in tFrsc-stimulated wells and medium wells for eight mice per group. Numbers of spleen tFrsc-specific cytokine-secreting T cells were measured by ELISpot. Data are expressed as the difference between the number of T cells in tFrsc-stimulated wells and medium wells. Data are shown as the average of three replicates for eight mice per group. Statistical differences between IgA^+^/^+^ and IgA^−^/^−^ groups were assessed following normality testing (Shapiro–Wilk). Normally distributed data were analyzed using an unpaired *t*-test, and non-normal data using a Mann–Whitney U-test. Differences were considered significant if *p* < 0.05. * *p* < 0.05; ** *p* < 0.01; *** *p* < 0.001; **** *p* < 0.0001; ns = non-significant.

Following RSV challenge, mice immunized with tFrsc/TriAdj showed significantly enhanced cytokine responses compared to PBS-treated animals. In tFrsc-restimulated lung cells, IL-17A levels were significantly higher in tFrsc/TriAdj-vaccinated IgA^−^/^−^ mice than in IgA^+^/^+^ mice (*p* < 0.05) (Figure 7a). While IL-17A levels did not differ between tFrsc/TriAdj-vaccinated and PBS-challenged IgA^+^/^+^ mice, they were significantly elevated in vaccinated IgA^−^/^−^ mice (*p* < 0.01), possibly indicating that tFrsc/TriAdj enhances local IL-17A production in the absence of IgA. IL-5 levels did not differ between genotypes following tFrsc/TriAdj vaccination, but were significantly higher in vaccinated IgA^−^/^−^ mice compared to their PBS-challenged counterparts (*p* < 0.05), whereas no such difference was observed in IgA^+^/^+^ mice. IFN-γ levels were comparable between vaccinated IgA^+^/^+^ and IgA^−^/^−^ mice, and neither group differed significantly from their respective PBS-challenged controls. In the supernatants of the splenocytes restimulated with tFrsc, vaccinated IgA^+^/^+^ and IgA^−^/^−^ mice produced significantly higher levels of IL-17A (*p* < 0.001), IL-5 (*p* < 0.001 and *p* < 0.01, respectively), and IFN-γ (*p* < 0.05 and *p* < 0.01, respectively) compared to PBS-immunized, RSV-challenged mice (Figure 7b). IFN-γ production was also increased in PBS-immunized, challenged mice of both genotypes but was absent in unchallenged animals, indicating that RSV infection is contributing to early IFN-γ induction. Analysis of cytokine-secreting T cells in the spleen showed significantly elevated numbers of IL-17^+^, IL-5^+^, and IFN-γ^+^ T cells in both tFrsc/TriAdj-vaccinated IgA^+^/^+^ and IgA^−^/^−^ mice compared to PBS-treated, challenged groups (*p* < 0.001 or *p* < 0.0001) (Figure 7c). However, no significant differences in cytokine-producing T-cell frequencies were observed between the two genotypes.

Recruitment of CD4^+^ effector T helper cells was assessed by flow cytometry following RSV challenge. 

**Figure 8:**
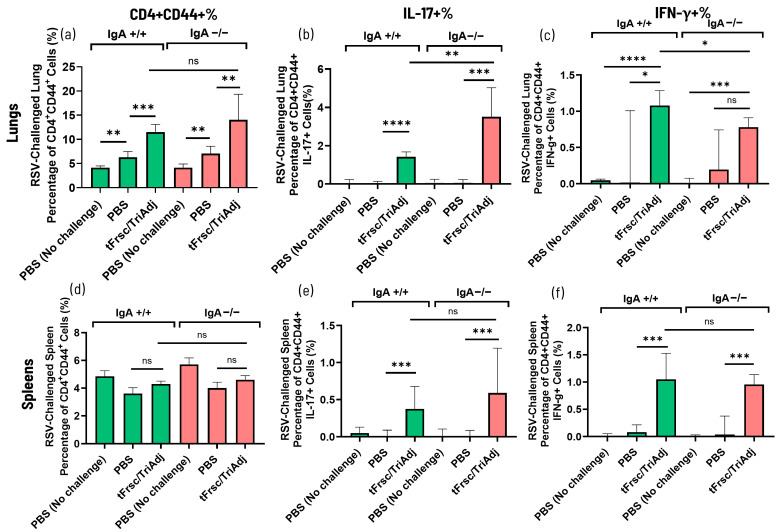
Numbers of effector cytokine-producing CD4+ helper T cells in the lung and spleen measured by flow cytometry in IgA^+^/^+^ and IgA^−^/^−^ BALB/c mice. Mice were immunized twice, three weeks apart, with PBS or tFrsc/TriAdj. Mice were IN challenged with 5 × 10^5^ PFU of RSV A2 strain three weeks post-secondary immunization and euthanized four days post challenge. Single-cell suspensions of spleen and lung cells were stimulated with tFsc protein. Cells were stained with viability dye and antibodies specific for CD4 and CD44, followed by fixation and permeabilization of the cells and incubation with antibodies specific for IL-17A and IFN-γ. Cells were gated for lymphocytes, singlets, and live cells, and then analyzed for the indicated markers. Results are expressed as the difference between the percentage of IL-17A^+^CD4^+^CD44^+^ or IFN-γ^+^CD4^+^CD44^+^ T cells in tFrsc protein-stimulated wells and the percentage of IL-17A^+^CD4^+^CD44^+^ or IFN-γ^+^CD4^+^CD44^+^ T cells in medium-stimulated wells. Activated lung CD4+CD44+ (**a**) and IL-17+ (**b**) or IFN-γ+ (**c**) T cells and activated spleen CD4+CD44+ (**d**) and IL-17+ (**e**) or IFN-γ+ (**f**) T cells are shown. Data are presented as median with interquartile range. Statistical differences between IgA^+^/^+^ and IgA^−^/^−^ groups were assessed following normality testing (Shapiro–Wilk). Normally distributed data were analyzed using an unpaired *t*-test, and non-normal data using a Mann–Whitney U-test. Differences were considered significant if *p* < 0.05. * *p* < 0.05; ** *p* < 0.01; *** *p* < 0.001; **** *p* < 0.0001; ns = non-significant.

In the lungs, tFrsc/TriAdj vaccination significantly increased the total CD4^+^ T cell numbers compared to PBS treatment (Figure 8a). IL-17^+^ T cell recruitment was also markedly enhanced in both vaccinated IgA^+^/^+^ (*p* < 0.0001) and IgA^−^/^−^ (*p* < 0.001) mice relative to PBS-treated, challenged animals, with a more pronounced response in IgA^−^/^−^ mice compared to their IgA^+^/^+^ counterparts (*p* < 0.01) (Figure 8b). Similarly, the IFN-γ^+^ T cell numbers were significantly increased in tFrsc/TriAdj-vaccinated IgA^+^/^+^ mice compared to PBS-challenged animals (*p* < 0.05). In contrast, vaccinated IgA^−^/^−^ mice did not show a significant increase in IFN-γ^+^ T cell numbers compared to PBS-challenged IgA^−^/^−^ mice, though levels were elevated relative to PBS-treated, unchallenged mice (*p* < 0.001) (Figure 8c). Notably, IFN-γ^+^ T cell frequencies in vaccinated IgA^−^/^−^ mice remained significantly lower than those in vaccinated IgA^+^/^+^ mice (*p* < 0.05). Collectively, these findings support impaired Th1 responses and suggest a skewing toward Th17 responses in the absence of IgA. In the spleen, total effector CD4^+^ T cell frequencies were low and did not differ between tFrsc/TriAdj-vaccinated and PBS-treated groups (Figure 8d). However, both IL-17^+^ and IFN-γ^+^ T cell frequencies were significantly increased in vaccinated IgA^+^/^+^ and IgA^−^/^−^ mice compared to the PBS-treated, RSV-challenged mice (*p* < 0.001), with no significant differences between genotypes (Figure 8e,f). Nonetheless, in the spleen, there tended to be an increase in IL-17^+^ and a reduction in IFN-γ^+^ T cell frequencies in IgA^−^/^−^ mice, mirroring the pattern seen in the lungs. Together, these findings suggest altered Th cell numbers with increased numbers of Th17 cells in IgA^−^/^−^ mice, particularly in the lungs.

#### 3.2.4. tFrsc/TriAdj Confers Lung but Not Nasal Protection in IgA-Deficient Mice

In the previous section, we observed that RSV was cleared from the lungs of all tFrsc/TriAdj-vaccinated mice, but persisted in the nasal passages of IgA^−^/^−^ mice. To further confirm that the protection conferred by tFrsc/TriAdj in IgA^−^/^−^ mice is restricted to the LRT, we assessed viral replication in the bronchoalveolar space using BAL samples. 

**Figure 9:**
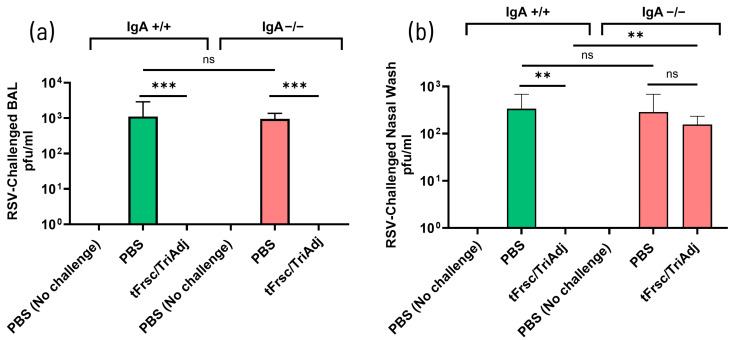
Virus replication in the lungs and nasal tissues of IgA^+^/^+^ and IgA^−^/^−^ BALB/c mice. Mice were IN immunized twice, three weeks apart, with PBS or tFrsc/TriAdj, followed by IN inoculation of 5 × 10^5^ PFU of RSV A2 strain three weeks post-secondary immunization and euthanasia four days post-challenge. Viral titers were determined in BAL (**a**) and nasal washes (**b**). Each bar represents the median RSV titer per milliliter of BAL or nasal wash for eight mice per group. Data were presented as median with interquartile range. Statistical differences between IgA^+^/^+^ and IgA^−^/^−^ groups were assessed following normality testing (Shapiro–Wilk). Normally distributed data were analyzed using an unpaired *t*-test, and non-normal data using a Mann–Whitney U-test. Differences were considered significant if *p* < 0.05. ** *p* < 0.01; *** *p* < 0.001; ns = non-significant.

Following the RSV challenge, no virus was detected in the lungs of IgA^+^/^+^ or IgA^−^/^−^ vaccinated mice, while PBS-treated animals exhibited high RSV titers (*p* < 0.001) (Figure 9a). RSV was completely cleared from the nasal passages of tFrsc/TriAdj-immunized IgA^+^/^+^ mice (*p* < 0.01 compared to PBS), while similar viral loads were present in the nasal passages of the PBS- and tFrsc/TriAdj-immunized IgA^−^/^−^ mice (Figure 9b). These findings corroborate the results from earlier trials and reinforce the essential role of nasal IgA in mediating protection in the URT.

## 4. Discussion

In this study, we examined mucosal and systemic humoral immune responses, T cell activation, and protection induced by IN immunization with tFrsc/TriAdj in wild-type and IgA-deficient BALB/c mice.

Vaccination with tFrsc/TriAdj induced robust IgG production in both systemic and mucosal compartments of IgA^+^/^+^ and IgA^−^/^−^ mice. However, nasal anti-tFrsc IgG was reduced, and anti-tFrsc IgG2a was decreased in the nasal wash, lungs, and sera of IgA^−^/^−^ mice. The reduction in total nasal IgG likely reflects the loss of this subclass, which may constitute a substantial fraction of IgG in the nasal mucosa. Consistent with this, studies in humans with selective IgA deficiency (SIgAD) showed that IgA deficiency is frequently associated with IgG subclass imbalances and compensatory patterns [39].

The absence of nasal IgA was associated with impaired URT protection following RSV challenge, indicating that IgG alone, at least at the titers induced here, might be insufficient for control of RSV replication in the URT, where IgA typically provides frontline defense by preventing viral adherence and entry [8]. IgA^−^/^−^ mice did not develop virus-neutralizing antibodies in the URT after immunization with tFsc/TriAdj, which is likely related to the absence of IgA, and might account for the significantly reduced protection from RSV infection. Indeed, Mills et al. showed that low nasal wash neutralizing titers are a stronger correlate of infection than serum titers [12]. Our data align with human RSV challenge studies. Habibi et al. reported that serum antibodies correlated poorly with URT protection, whereas higher nasal RSV-specific IgA nearly doubled the likelihood of protection [17]. Similarly, adults with elevated nasal IgA were less likely to become reinfected, with nasal IgA levels correlating more strongly to reduced viral load than serum IgA [11,17]. Importantly, nasal IgA is predominantly polymeric, existing mainly as dimers composed of two IgA monomers linked by the J chain and associated with the secretory component. These structural features enhance proteolytic resistance and likely contribute to IgA’s superior ability to neutralize RSV at the mucosal surface [40]. Walsh et al. also identified low nasal IgA as a significant risk factor for RSV infection in adults, although they noted that both serum and nasal antibodies contribute to protection [10]. Pierantoni et al. reported that a single IN or intramuscular dose of MVA-RSV completely protected BALB/c mice and cotton rats from RSV replication in the lungs, whereas only IN administration prevented infection in the URT [41]. Taken together, these findings indicate that, while IgA is not the sole protective mechanism at play, its localized induction is a critical component of URT protection.

In contrast to the URT, both IgA^+^/^+^ and IgA^−^/^−^ mice achieved complete viral clearance in the lungs and BAL following tFrsc/TriAdj vaccination. The IgA^+^/^+^ and IgA^−^/^−^ mice also generated similar RSV-specific neutralizing antibody titers in serum and lungs, indicating that pulmonary anti-tFrsc IgG-secreting cells and/or transudated IgG were likely sufficient to achieve complete viral suppression in the LRT in the absence of IgA. Indeed, murine studies suggest that nasal and lung immunity are compartmentalized [42]. The contention that IgA is indispensable for protection of the nasal mucosa, whereas plasma-derived IgG safeguards the lungs, has been proposed as a consequence of “mucosal leakiness” in the LRT [43,44]. IgG’s dominance in the lungs and IgA’s prominence in the nose are supported by influenza models; ~90% of influenza-specific RT IgG was concentrated in the lungs as the dominant protective antibody, while only ~6% was present in nasal mucus, where most RT IgA was located [45,46]. Our findings therefore align with previous studies showing that vaccine-induced IgA is essential for URT protection against viral infection, but largely dispensable in the lower airways.

Splenic anti-tFrsc IgG^+^ B cell numbers were reduced in tFrsc/TriAdj-immunized IgA^−^/^−^ mice, representing a novel aspect of this work and suggesting that IgA deficiency may selectively impair systemic B cell compartmentalization or maintenance. Our observations are consistent with compromised generation, survival, or retention of IgG^+^ B cells in secondary lymphoid organs in the absence of IgA. The preservation of high serum IgG and neutralizing antibody titers alongside reduced splenic IgG^+^ B cells implies that IgG production may rely predominantly on plasma cells originating outside the spleen. IgA may indirectly support systemic B cell differentiation through immune network effects, including modulation of mucosal antigen presentation. IgA–antigen complexes translocated into the subepithelial dome region via M cells can be captured by CX3CR1^+^ DCs through receptors such as DC-SIGN [47,48]. Similarly, a subset of human DCs expresses the IgA Fc receptor CD89 (FcαRI), which can be engaged by IgA immune complexes, leading to receptor-mediated internalization, activation of DCs, and amplification of the production of pro-inflammatory cytokines [49,50]. Such DCs later associate with CD4+ T cells in the interfollicular region and help regulate both immune tolerance and adaptive immune responses [51,52]. Supporting this, IgA^−^/^−^ mice immunized IN with influenza vaccine exhibited a depressed splenic lymphocyte proliferative response to phytohemagglutinin, suggesting a role for IgA in facilitating antigen presentation to T cells [53]. Additionally, previous work in SIgAD patients has revealed features of gastrointestinal immune dysregulation and systemic immune alterations, including elevated inflammatory cytokines and hyperactivated CD8^+^ T cells [54]. Additionally, dysregulated T follicular helper and T follicular regulatory cell responses, important regulators of germinal center (GC) reactions and B cell differentiation, have been reported in the context of IgA-deficient mice [55]. These studies reveal that mucosal IgA deficiency may lead to aberrant immune responses, which may lead to immune dysregulation and might possibly contribute to the impaired splenic IgG^+^ B cell frequencies observed in our IgA^−^/^−^ mice.

Since we observed a reduction in splenic IgG^+^ B cell frequencies in tFrsc/TriAdj-immunized IgA^−^/^−^ mice, GC activity in the spleen may be compromised in the absence of IgA, which could impair the formation of high-affinity, class-switched memory B cells or long-lived plasma cells. However, we did not assess GC activity in secondary lymphoid tissues, which limits our understanding of long-term immunity following vaccination, particularly in relation to the durability of protection and susceptibility to reinfection in IgA^−^/^−^ mice. These aspects will be addressed in future studies.

In contrast, IgG^+^ B cell numbers in the lungs were comparable between IgA^+^/^+^ and IgA^−^/^−^ mice after tFrsc/TriAdj vaccination, suggesting that the local response is preserved in the absence of IgA. Interestingly, Arnaboldi et al. reported defective lung B cell expression in IgA^−^/^−^ mice in a model of allergic inflammation without adjuvant, while in other studies, IgA^−^/^−^ mice immunized with an influenza subunit vaccine resulted in loss of protection unless a potent DC-stimulating mucosal adjuvant was included [31,53,56]. These observations suggest the importance of the use of adjuvants in compensating for the absence of IgA. The PCEP component of tFrsc/TriAdj may have played a central role in what appears to be “masking” the need for IgA in the lower airways, as it can recruit DCs, enhance antigen uptake and presentation, and stimulate local antigen-specific inflammatory responses [37,57,58]. Following IN tFrsc/TriAdj immunization, these effects may have facilitated localized DC activation and maturation in the lungs, enabling robust anti-tFrsc B cell responses. Therefore, our results suggest that IgA production may influence the regulatory mechanisms that sustain appropriate B cell homeostasis, while its effect in the LRT might be substituted by potent mucosal DC-activating adjuvants through IN immunization. Thus, IN vaccination with tFrsc/TriAdj induced LRT protection, underscoring that, while IgA is indispensable for URT protection, it may not be strictly required in the LRT if supported by strong immunostimulatory adjuvants.

IgA^−^/^−^ mice consistently showed reduced IgG2a and either comparable or elevated IgG1 levels following tFrsc/TriAdj vaccination and RSV challenge. In addition, after vaccination with tFrsc/TriAdj, IgA^−^/^−^ mice displayed elevated IL-17 production in some contexts, as well as enhanced numbers of IL-17^+^ CD4^+^ T cells, with a concomitant decrease in Th1-type IFN-γ^+^ T cells compared to IgA^+^/^+^ mice in the lungs, which is a novel finding of this study. Th17 CD4^+^ T cells are a major source of IL-17, although other populations such as ILCs and NK cells may also produce IL-17. However, as enhanced IL-17 production was only induced in tFsc/TriAdj-immunized mice, this likely reflects an adaptive response mediated by Th17 cells. This would have to be confirmed by transcription profiling. Meanwhile, IL-5 levels and Th2-type IL-5^+^ T cell frequencies were similar across genotypes. These findings parallel those of Zhang et al., who observed significantly reduced IFN-γ, but comparable IL-4 and IL-5 responses in IgA^−^/^−^ after IN vaccination with influenza subunit and viral challenge [32].

## 5. Conclusions

In summary, our data demonstrate that IN immunization with tFrsc/TriAdj elicits strong systemic and pulmonary immunity in both wild-type and IgA-deficient mice, but fails to protect the URT in the absence of IgA. IgA deficiency was associated with reduced IgG2a levels, possibly supporting an attenuation of Th1 polarization and what may be a Th17 bias. These findings suggest that IgA contributes to supporting Th1-type immunity and shaping the overall quality of vaccine-induced responses. Importantly, robust induction of mucosal IgA appeared to be indispensable for complete protection against RSV and for limiting Th2-skewed environments that may predispose to immunopathology. Taken together, these results provide mechanistic insights to guide the development of future mucosal vaccination strategies against RSV.

## Data Availability

Data presented in this study are available on request.

## Supplementary Materials

The following supporting information can be downloaded at: https://www.mdpi.com/article/10.3390/vaccines14010097/s1. Supplementary Figure S1 shows the progression of serum RSV tFrsc-protein-specific antibody responses to immunization, while Supplementary Figure S2 shows the progression of serum RSV tFrsc-protein-specific antibody responses following immunization and RSV challenge.
