# Supplementary figures and images for: Evaluation of a Respiratory Syncytial Virus Subunit Vaccine Candidate in IgA-Deficient Mice: Insights into the Role of IgA in Vaccine-Induced Immunity and Protection
Source: Vaccines (Basel). 2026 Jan 20;14(1):97. doi: 10.3390/vaccines14010097 (PMC12846617; doi:10.3390/vaccines14010097)

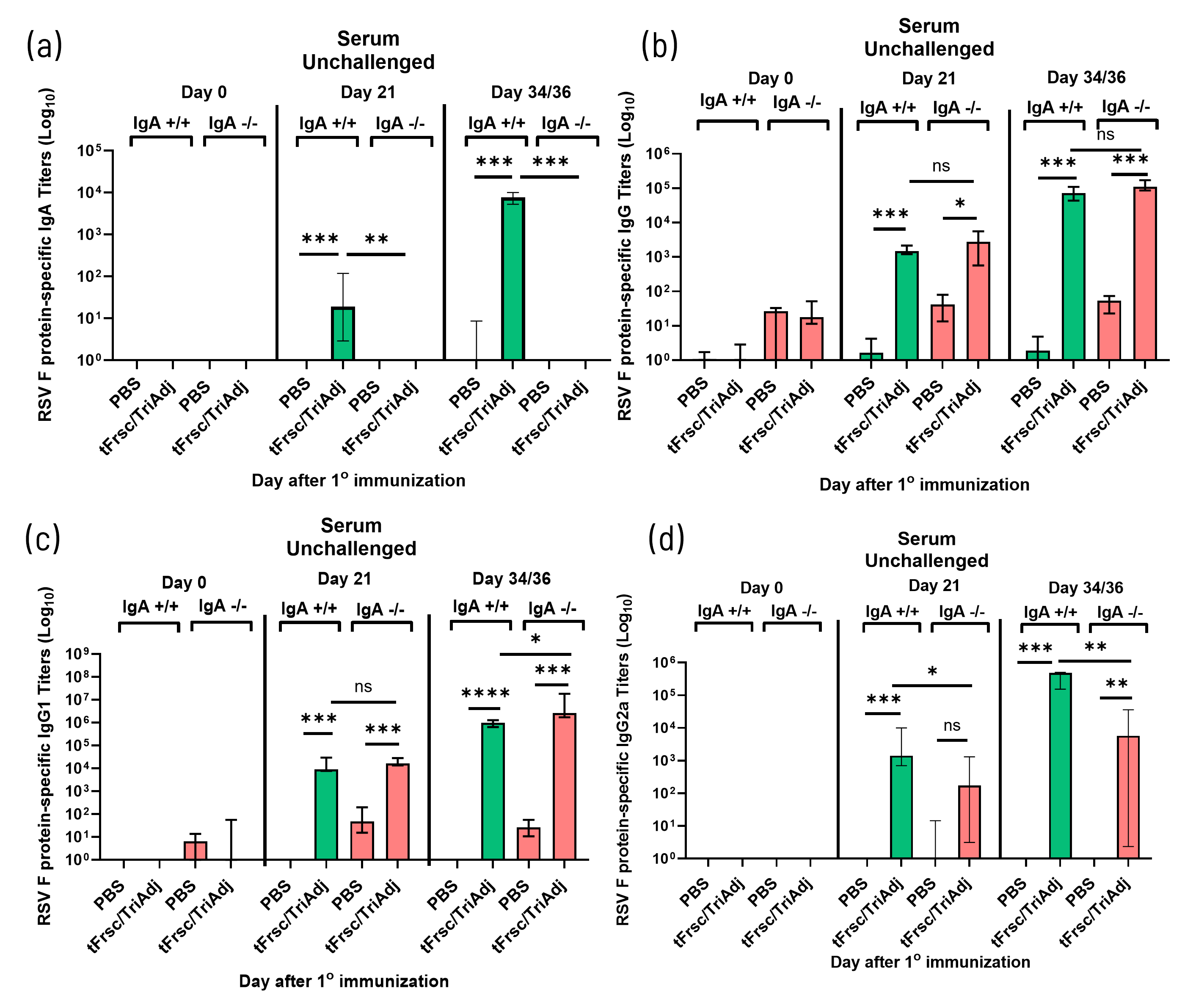

Supplement: Supplementary file 1 [file vaccines-14-00097-s001.zip › supp fig 1.png]

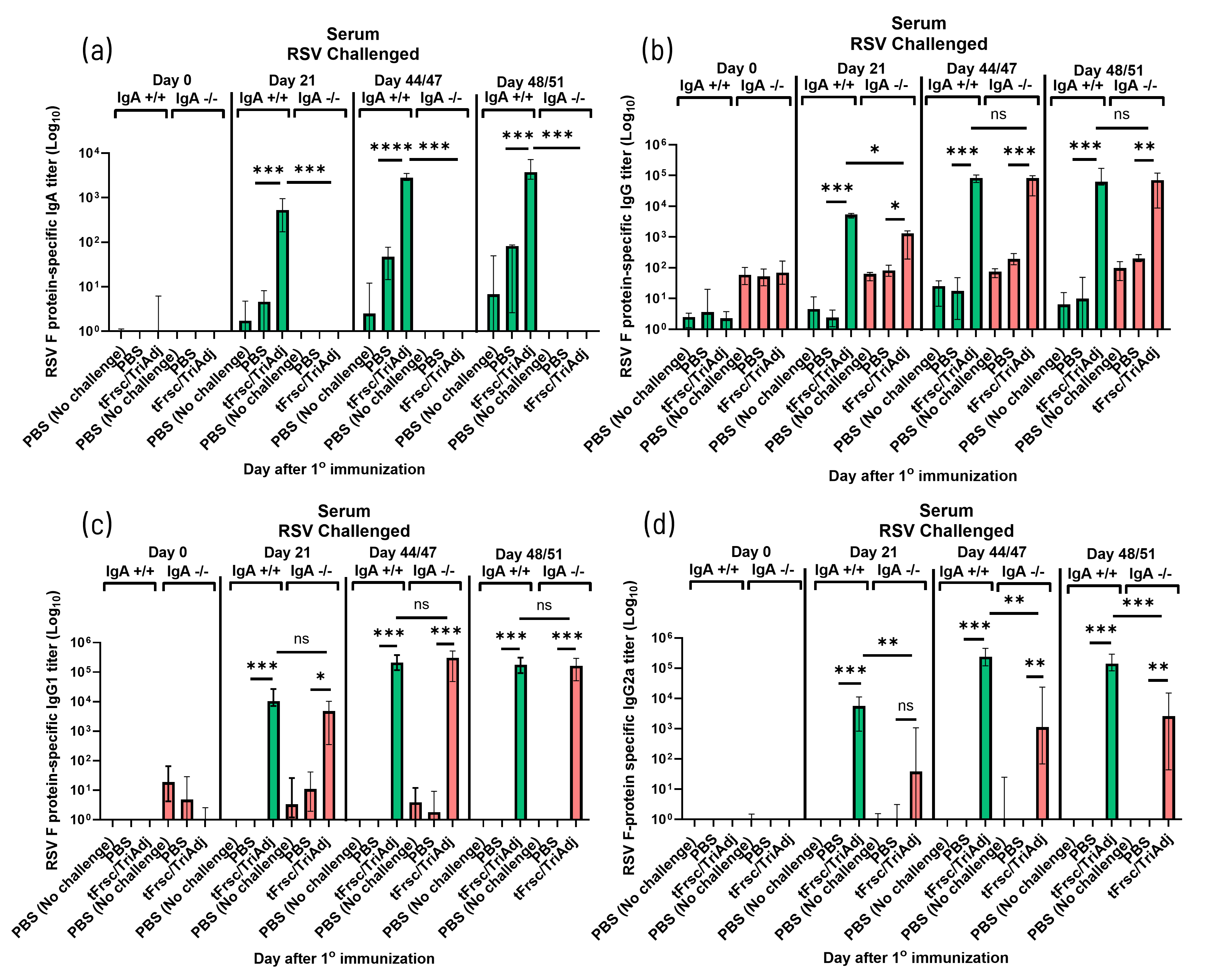

Supplement: Supplementary file 1 [file vaccines-14-00097-s001.zip › supp fig 2.png]
